# Impacts of an Amazonian hydroelectric dam on frog assemblages

**DOI:** 10.1371/journal.pone.0244580

**Published:** 2021-06-17

**Authors:** Jussara Santos Dayrell, William Ernest Magnusson, Paulo Estefano Dineli Bobrowiec, Albertina Pimentel Lima

**Affiliations:** 1 Programa de Pós-Graduação em Ecologia, Instituto Nacional de Pesquisas da Amazônia, Manaus, Amazonas, Brazil; 2 Coordenação de Biodiversidade, Instituto Nacional de Pesquisas da Amazônia, Manaus, Amazonas, Brazil; University of South Carolina, UNITED STATES

## Abstract

About 90% of the Amazon’s energy potential remains unexploited, with many large hydroelectric dams yet to be built, so it is important to understand how terrestrial vertebrates are affected by reservoir formation and habitat loss. We investigated the influence of the construction of the Santo Antônio Hydroelectric dam on the Madeira River in southwestern Amazonia on the structure of frog assemblages based on samples collected in two years before the dam flooded (pre-stage) and one (post1-stage) and four years (post2-stage) after its construction. We surveyed five 500-ha plot systems three times during each stage; in the pre-stage we sampled 19 plots in low-lying areas that would be flooded by the dam, (from now called flooded pre-stage plots) and 45 plots in terra-firme forest (from now called unflooded pre-stage plots). At the post1-stage we sampled the 45 unflooded plots and in the post2-stage we sampled the remaining 39 unflooded plots. We detected frogs by active visual and acoustic searches standardized by both time and sampling area. Few species recorded in the pre-stage flooded plots were not found in the pre-stage unflooded plots or in stages after flooding. However, the composition of frog assemblages based on relative densities in flooded pre-stage plots did not re-establish in plots on the new river margins. In unflooded areas, frog assemblages were distinct among the flooding stages with no tendency to return to the original assemblage compositions even four years after the dam was filled. For the areas that were not flooded, there was an increase in species richness in 82% of the plots between the surveys before dam construction and the first surveys after dam completion, and 65% between the pre-stage and surveys four years after dam completion. Lack of understanding by the controlling authorities of the long-term effects of landscape changes, such as water-table rises, means that studies covering appropriate periods post construction are not required in legislation, but the data from Santo Antônio indicate that changes due to dam construction are either long-term or difficult to distinguish from natural fluctuations. Future environmental-impact studies should follow strict BACI designs.

## Introduction

One of the factors contributing most to deforestation and consequent biodiversity loss in the Amazon is implementation of government infrastructure programs, such as the construction of hydroelectric dams on large rivers [1]. Currently, 256 of the 412 large hydroelectric dams in operation, being constructed or planned for the Amazon are in Brazil [2]. Large hydroelectric dams in operation up to 2012 flooded 1,105,400 ha of forests in the Amazon [3], and can affect aquatic and terrestrial biodiversity [4,5].

Damming rivers floods large forest areas, reduces the flood pulse and changes water-table depth [6,7]. In addition to reducing flood pulses, damming reduces várzea forest, a type of seasonally-flooded forest with unique fauna [8–13] and flora [14,15]. Várzeas are flooded annually by nutrient-rich white-water rivers for 6 to 8 months and these highly-diverse areas connect habitats and play a key role in maintaining regional biodiversity due to their spatial and temporal complexities [16,17]. After dam construction, lowland vegetation, including várzea forest, is permanently inundated and the ground-water level in the unflooded area is raised, modifying the original vegetation [18]. The creation of new habitats can influence the abundance and distribution of terrestrial species associated with humid lowlands, but the extent of the effects of the new habitat type on the unflooded area is unknown.

The Madeira River is the main tributary of the Amazon River [19] and the fifth largest river in the world in terms of water flow [20]. The Madeira River sub-basin is one of the most endangered in the Amazon, as 40 hydroelectric dams are already operating or under construction in the sub-basin [2]. In 2011, two large dams on the Madeira River had their construction completed (Santo Antônio and Jirau; ~ 3500 MW each). These are run-of-the-river dams whose turbines use the river current to generate hydroelectricity and are generally considered less harmful to the environment than conventional hydroelectric dams since they flood smaller areas and generally do not form islands [21]. However, there are no published data on the impacts of run-of-the-river dams on anurans in the Amazon.

Anurans are sensitive to changes in the environment [22] and climatic [23] variations due to permeable skin, dependence on humid environments for reproduction and biphasic life cycle [24–26]. Hydroelectric dams modify water levels and water availability, affecting terrestrial and riparian habitats and the amphibian species that occupy them [6,27,28]. In addition, the structure of riparian forests affects assemblages of leaf-litter frogs [29], and distance to and availability of water bodies are important for species with aquatic reproduction [30].

With the growing number of hydroelectric dams planned for the Brazilian Amazon [2], it is important to understand how vertebrate assemblages respond to habitat modification and loss. Several studies have shown that frogs are sensitive to such changes [31], and long-term monitoring with sampling before and after the dam floods is an opportunity to understand the temporal and spatial effects of dam construction.

We investigated the influence of the construction of the Santo Antônio Hydroelectric dam on the structure of frog assemblages based on samples collected in three-time intervals (two years before the dam flooded [pre-stage], and one [post1-stage] and four years [post2-stage] after its construction). The sampling regime was defined by the national environmental agency (IBAMA) and did not follow a Before-After-Control-Impact (BACI) design [32] as it did not include a control area. Nevertheless, it permits a general evaluation of changes in the anuran assemblages in that period.

We asked the following questions: 1) Did flooding result in the loss of species in the region around the dam?; 2) Did frog assemblages from flooded areas reestablish on the new river banks after dam construction?; 3) Did frog assemblages in the unflooded plots change in ways that might suggest that they were affected by flooding of adjacent areas?; 4) If there were changes in the frog assemblages in unflooded areas, did these changes occur immediately after the construction of the dam or with a delay?; 5) Were any such changes greater in areas near the reservoir than in areas further away?.

We hypothesized that the frog assemblages in periodically flooded lowland forests (várzea) would be eliminated and that the species composition found in this and other vegetation associations inundated by the dam might not reestablish if the habitat on the new banks of the river after dam construction did not resemble that in the areas that were inundated. We also predicted that the species composition of the unflooded areas would change over time, especially the areas near the new banks of the Madeira River and its tributaries where the water table would be elevated.

## Material and methods

### Study area

The Madeira River is one of the main tributaries of the Amazon River, being responsible for 15% of the discharge of the Amazon into the Atlantic Ocean [33]. Its waters are turbid due to suspended sediments derived from the Andes [34]; it is the most sediment-laden river in the Amazon Basin [35].

The predominant vegetation type in the upper Madeira River region was originally composed of dense tropical rainforests, with a mosaic of terra-firme forest, várzea on the river banks and patches of white-sand vegetation locally called “campinarana” [36]. According to the Köppen classification, the predominant climate is Aw—Tropical Rainy. The average annual temperature varied from 25° to 27°C and the annual precipitation between 1400 and 2000 mm between 1998 and 2007 (data from the National Water Agency, ANA). The dry season generally occurs from June to September and the rainy season from November to April, with precipitation >330 mm per month in December and January. River levels can vary by more than 12 meters in some parts of the upper Madeira River [37].

The Santo Antônio Hydroelectric dam (08°48’S; 63°57’W) is located 10 km upstream of the city of Porto Velho, Rondônia state, and is the fourth largest hydroelectric dam in operation in Brazil with 3,150 MW of installed capacity [1]. It has been in operation since March 2012 with the water level at 70.2 m above the original river level. The bulb-type turbines require less water, producing a reduced reservoir size (271 km^2^) than conventional Amazonian hydroelectric dams, such as Tucuruí, Balbina and Samuel [1].

### Sampling design

**Fig 1:**
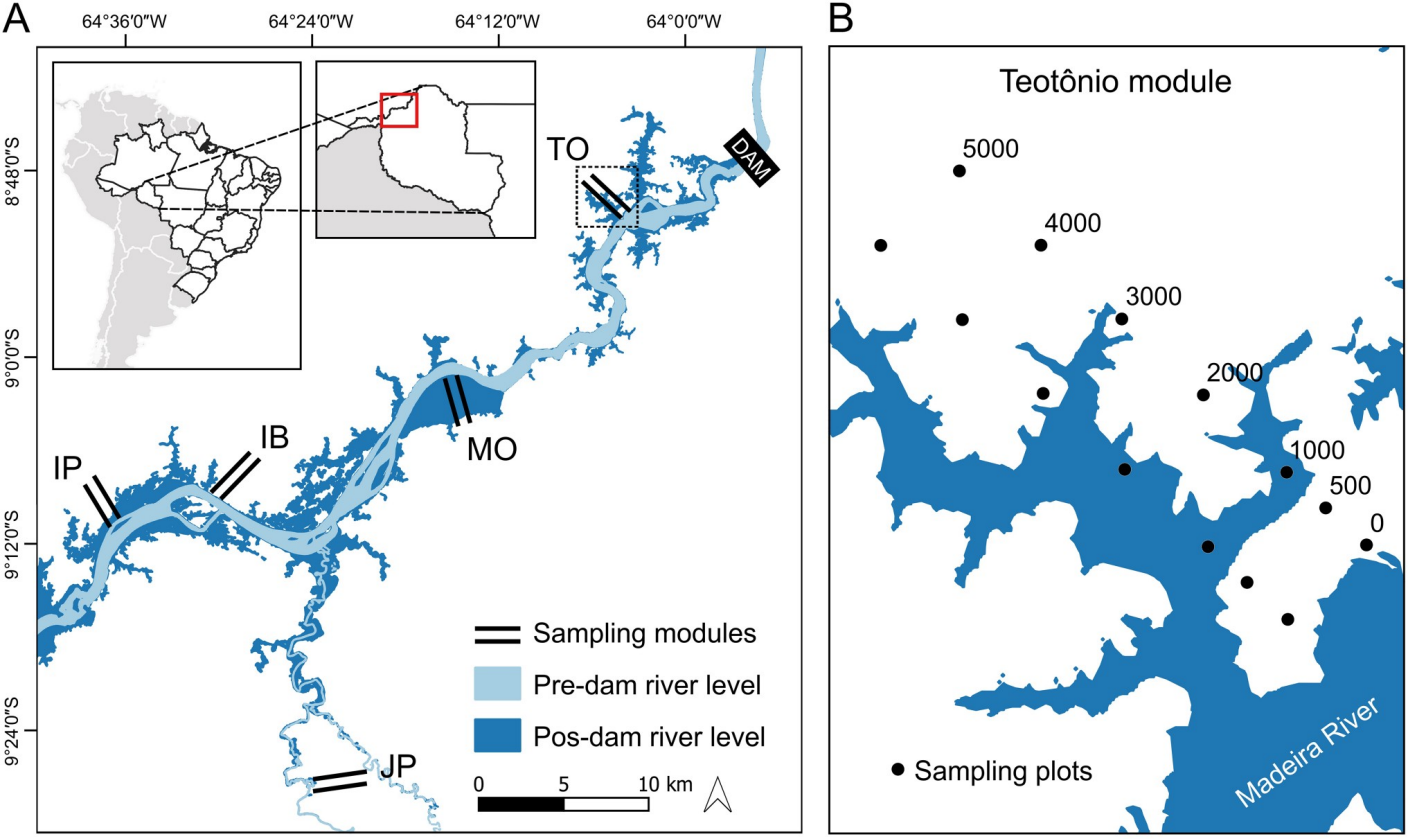
Location of the study area along a 100-km section of the Madeira River showing the five sampling modules. TO = Teotônio, MO = Morrinhos, IB = Ilha de Búfalos, IP = Ilha das Pedras, JP = Jaci Margem Direita. In detail (right), design of modules with two 5 km trails and seven plots (black circles) distributed 0, 500, 1000, 2000, 3000, 4000, and 5000 m from the original bank of the Madeira River.

Five sampling modules were installed from 10 to 100 km upstream of the dam as part of a government-mandated impact assessment. The location of each module was chosen by the environmental authority, taking into account the presence of enough vegetated area for the installation of the modules, as this region is extremely deforested. The configuration of the modules followed the method of biodiversity survey (RAPELD) developed by the Biodiversity Research Program (PPBio) [38]. Each module consisted of two parallel 5 km trails perpendicular to the Madeira River, separated by 1 km. Seven 250 m long plots were installed along each trail (14 plots per module). The center lines of plots followed the contours of the terrain to minimize within-plot topographic and vegetation variation. Plots were established at distances of 0, 500, 1000, 2000, 3000, 4000 and 5000 m from the original river bank (before flooding). Three modules were on the left bank of the Madeira River (Teotônio, Ilha de Búfalos, Ilha das Pedras), one was on the right bank (Morrinhos) and one was on the right bank of the Jaci-Paraná River (Jaci Margem Direita), a tributary of the right bank of the Madeira River (Fig 1).

The modules were sampled three times during each of the following stages: pre-filling (pre-stage) two years before the dam was filled in February 2010, November 2010 and February 2011; post-filling 1 (post1-stage) for surveys undertaken one year after dam filling, in February 2013, November 2013 and February 2014; post-filling 2 (post2-stage), which occurred four years after dam filling, in November 2016, January 2017 and February 2017. In the pre-stage, 64 plots were sampled. Two plots in Ilha Búfalos module, two in Morrinhos and one in Teotonio were not sampled for logistical reasons. The Jaci Margem Direita module has 13 plots. Of the 64 plots surveyed in the pre-stage, 19 were permanently flooded after the construction of the dam, and we refer to them as pre-stage flooded. The 45 unflooded plots sampled before the construction of the dam we refer to as pre-stage unflooded. These were also sampled in the post1-stage, but in 2016 six plots were deforested and only 39 remained in the post2-stage samples (S1 Table).

### Frog sampling

We sampled the frogs by visual and acoustic survey limited by time and space along the sampling plot (250 x 10 m) with two observers per plot. To detect frog species with different activity periods (diurnal, crepuscular and nocturnal), visual and acoustic surveys were conducted between 16:30 and 18:30, and between 19:00 and 23:00.

We recorded the presence or absence of each species in each 10 m section of the 250 m long plot, resulting in a relative-abundance index that varied between 0 and 25 records per species in each plot. This standardization was necessary because some species (e.g. *Adenomera* spp., *Phyzelaphryne* spp., *Pristimantis* spp.) have high densities of calling males during the reproductive period [39], which makes it difficult to estimate the number of individuals, and some subterranean and leaf-litter species were only detected by calls and could not be counted directly.

The survey teams collected a maximum of three voucher specimens per species, per plot. These were anesthetized and euthanized with 5% xylocaine, fixed in 10% formalin, preserved in 70% ethanol and deposited in the herpetology section of the INPA Zoological Collection in Manaus, Amazonas, Brazil. All were identified in the laboratory with the help of specialized guides (e.g. [39–43]).

In Brazil, the collection or transport of biological material for scientific or teaching purposes requires authorization by the System for Authorization and Information on Biodiversity (Sisbio). This system is administered by the National Institute of Environment and Renewable Natural Resources (IBAMA), which is responsible for the ethical treatment of animals. Frogs were collected as part of government-mandated environmental assessment surveys, under IBAMA/SISBIO (Ministry of Environment, Government of Brazil) permit No 13777–2. This permit was subject to approval of all ethical procedures for catching and collecting species and specimens. We followed the directives of the Federal Council for Biology (CFBIO) Resolution CFBIO N° 08/12/2012, which relates to procedures for capture, containment, release and collection of vertebrates in situ and ex situ.

### Analyses

We used sample-based rarefaction (interpolation) and extrapolation curves with 95% unconditional confidence intervals [44] to compare total frog richness between and within flooding stages. Richness and interpolation (rarefaction) and extrapolated curves of pre-stage flooded (n = 19 plots), pre-stage unflooded (n = 45), post1-stage (n = 45) and post2-stage (n = 39) were generated using the “iNEXT” package [45].

To evaluate the effect of dam construction on the composition of frog species in a bidimensional space and represent the sampled sites in different temporal stages, we used ordinations by Principal Coordinates Analysis (PCoA) based on the Bray-Curtis dissimilarity index for relative-abundance data and the Jaccard index for occurrence data. However, distance-based analyses have been shown to confound trends in location with changes in dispersion, leading to potentially misleading results [46]. Therefore, we also used a latent-variable model-based ordination implemented in the boral program (Bayesian ordination and regression analysis) [47] which uses Another Gibbs Sampler (JAGS) [48]. The Bayesian model-based approach accounts for the increasing mean-variance relationship without confounding location with dispersion [47]. However, the Boral did not converge for one of the analyses, and the configurations produced by Boral (S5 and S6 Figs) were similar to those produced by PCoA in all the other analyses. Therefore, we used PCoA to describe the patterns in all analyses in the main text.

To assess changes in species structure over time in the unflooded area, we grouped all data in each survey period resulting in nine points based on the same 39 plots sampled in each survey period and used the PCoA ordinations based on the Bray-Curtis dissimilarity index for relative-abundance data.

To determine if flooding resulted in changes in species composition in the region flooded by the dam we compared the pre-stage flooded (n = 19 plots), pre-stage unflooded (n = 45), post1-stage (n = 45) and post2-stage (n = 39) plot categories using the multivariate extension of generalized linear models (manyglm) function [46] in the mvabund package [49,50]. This model-based approach allows for hypothesis testing, and unlike distance-based methods, does not confound location and dispersion effects due to the misspecification of the mean–variance relationship [46]. The effect of flooding on the assemblages was evaluated using the anova.manyglm function which resampled the fitted model using ‘pit-trap’ bootstrapping to resample abundance data while accounting for correlations among species. The p-value was calculated from 999 bootstraps. Pairwise comparisons between flooding categories were assessed using the option in the anova.manyglm function to assess whether the assemblages in the flooded areas (pre-stage flooded) were different from the other areas. We fitted a multivariate generalized linear model with flooding categories as the predictor variable. The response variables were abundance data analyzed using a negative-binomial distribution and occurrence data analyzed using a binomial distribution for mvabund analyses.

These analyses were used with the complete assemblage (96 species) but rare species could represent a source of noise in multivariate analyses and thus prevent the detection of patterns of assemblage structure [51] and some studies have not included them in the analyses [52,53]. Rarity is subjective, but we wanted to know whether patterns for species with few records in our sample were similar to those for more common species [52,54]. We assessed this potential effect by undertaking analyses both with the complete dataset and with only abundant species (at least 5% abundance and 4% of plots in our sample) (S3 Table) and only with species considered rare in the sampling (only records with up to 5% abundance and 4% of plots in our sample) (S4 Table). We repeated the same analyses changing using 8% abundance and 5% of plots in our sample (S5 Table). As the results were similar, we only present analyses using the whole data set in the main text.

Histograms of species distributions along environmental gradients [55] constructed in R [56] were used to describe responses of individual species in relation to flooding stages for both abundance and occurrence data.

To quantify the temporal gains and losses of species in unflooded plots between stages, we used the Temporal Beta Index (TBI) [57] in R [56]. We used the TBI function of the adespatial package [58] with the Bray-Curtis distance for relative-abundance data and the Jaccard dissimilarity index for species-occurrence data in each plot. TBI is used to compare the dissimilarity values of a plot at time 1 with the dissimilarity values of the same plot at time 2 and is composed of two parts: B = species losses (or losses in abundance per species) and C = species gains (or gains in abundance per species). We tested whether the plots were dominated by species gain or loss, and increase or decrease in species abundance, using paired t tests with 9999 permutations. As the TBI analysis compares pairs of plots, we used the same 39 plots sampled in the pre-stage unflooded, post1-stage and post2-stage categories to compare the pre-stage unflooded assemblages with those in post1 and post2-stages.

To assess the effect of distance from the bank after dam flooding on assemblage composition, we compared the species composition of the plots located between 0.35 to 2 km from the flooded areas with the plots between 2.2 and 5.0 km. As the volume of water in tributary streams increased permanently with the flooding of the dam, we used the shortest distance from the flooded area instead of the distance to the new Madeira River bank. Individuals of most frog species are unlikely to travel more than 2 km between breeding sites. We used the option of pairwise comparisons in the anova.manyglm function in the mvabund package described previously with frog-abundance data from plots sampled in all no-flood categories (n = 39) assuming a negative-binomial distribution of the data, and based on the same indices of similarity and standardization described previously to test the statistical significance of changes in species structure over time in the unflooded area (pre-stage unflooded, post1-stage and post2-stage) in both near (≤ 2 km) and distant (> 2 km) plots.

Analyses were conducted in R [56].

## Results

**Fig 2:**
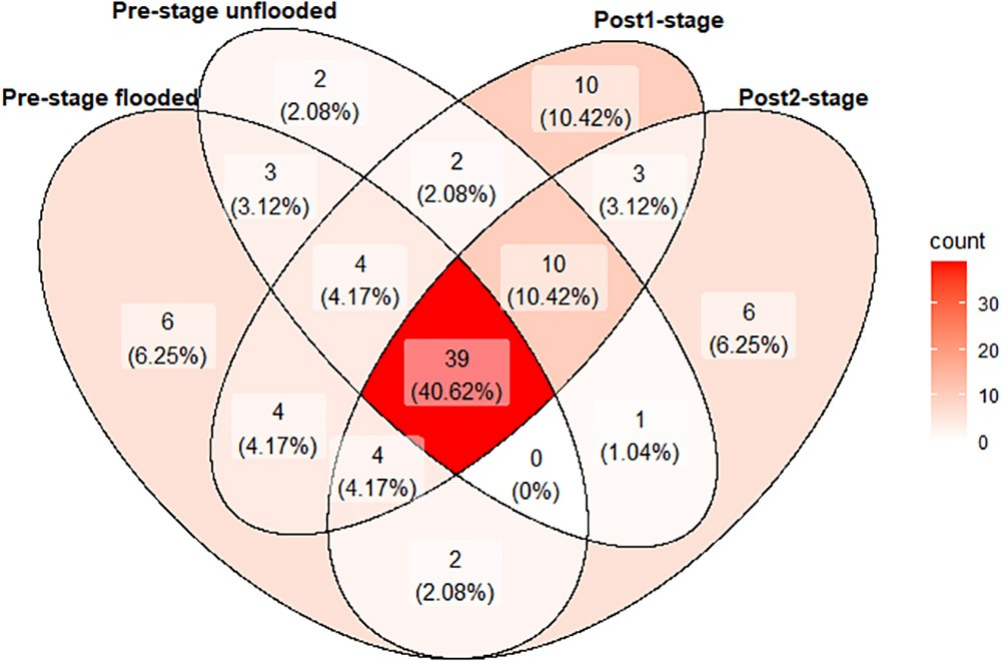
Venn diagram showing the overlap in species registered in the different combinations of flooding and time since filling of the Santo Antônio dam in the Madeira River, southwestern Brazilian Amazonia.

**Fig 3:**
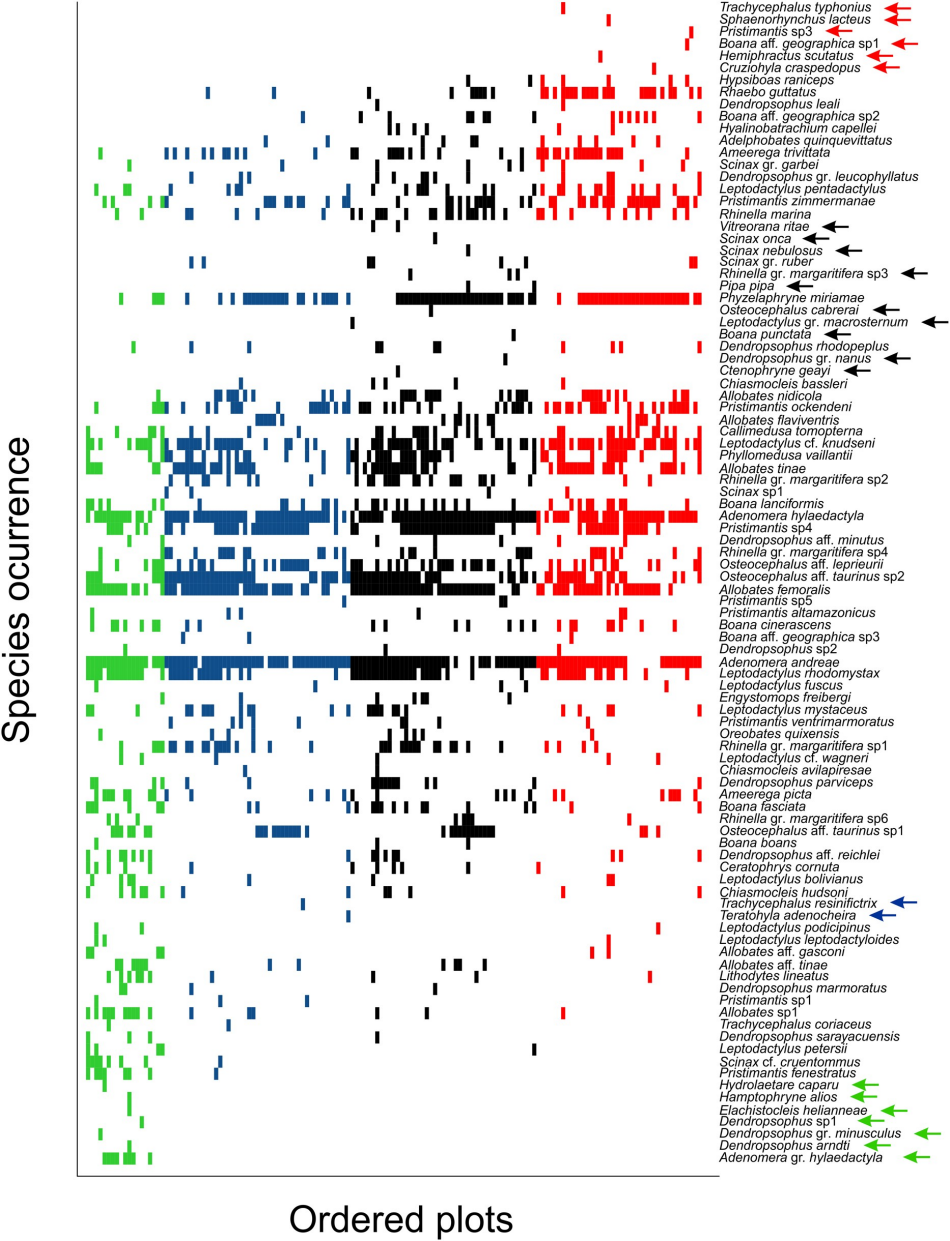
Plots of the occurrence of frogs in relation to flooding and time since filling of the Santo Antônio dam in the Madeira River, southwestern Brazilian Amazonia. green = plots that were sampled pre-filling that were flooded, blue = plots that were sampled pre-filling that were not flooded, black = plots sampled 1 year after dam filling, red = plots sampled 4 years after dam filling. Arrows indicate the species registered only in one flood-by-time category (indicated by color).

We recorded 96 species of frogs distributed in 32 genera and 13 families (S2 Table). We recorded 62 species before flooding in plots that would be inundated, and 61 species were recorded in unflooded plots in the pre-stage. We recorded 73 species in the post1-stage and 65 species in post2-stage, of which 39 (40.6%) occurred in all flooding stages, while nine were exclusive to before flooding, seven in plots that were flooded and two in unflooded plots. Ten species were found only in post1-stage and six only in post2-stage (Figs 2 and 3).

Based on the sample-based rarefaction curves, numbers of species detected per plot in the pre-stage flooded and post1-stage categories were higher than the number detected in the pre-stage unflooded and post2 unflooded categories. However, extrapolation to 60 plots indicates that the 95% confidence intervals converge, so flooding stages might not differ in the total number of species they support in the whole area, even though there are differences in the mean number of species per plot (S1 Fig).

**Fig 4:**
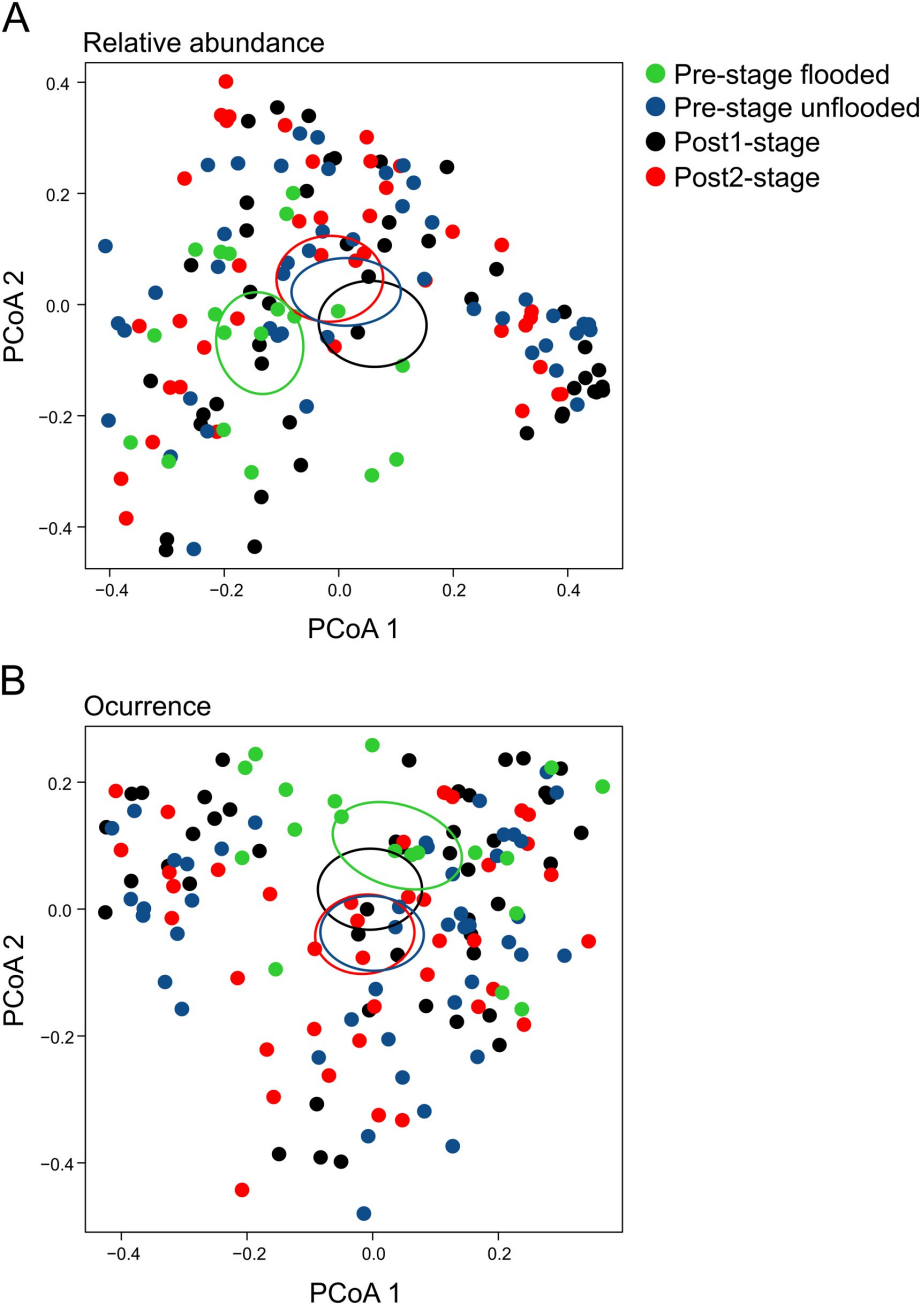
First two axes of a principal coordinates analysis (PCoA) based on relative abundance (A) and occurrence (B) of frog species showing the 95% confidence ellipses for the centroids of samples in plots in relation to inundation and time since filling of the Santo Antônio dam on the Madeira River, southwestern Brazilian Amazonia. green = plots that were sampled pre-filling that were flooded; blue = plots that were sampled pre-filling that were not flooded; black = plots sampled 1 year after dam filling; red = plots sampled 4 years after dam filling.

The multivariate general linear model (GLM) analyses indicated that the species compositions differed among the four categories of flooding by stage for the occurrence data (Wald = 19.14, p = 0.001) and for the relative-abundance data (Wald = 20.46, p = 0.001). Pairwise comparisons indicated that the species composition differed between all pairs of flooding categories (Table 1). The PCoA plots showed little difference in frog species composition of pre-stage unflooded, post1-stage and post2-stage, but pre-stage flooded plots were generally distinct (Fig 4).

**Table 1:** Manyglm analysis of the association between the structure of frog assemblages in flooded and unflooded plots around the Madeira River, southwestern Brazilian Amazonia in relation to inundation and time before or after dam filling.

**Overall effect—Abundance**	**Wald**	**P**
All categories	20.46	0.001
**Post hoc pairwise comparisons**	**Sum-of-LR statistic**	**P**
Pre-stage flooded vs. pre-stage unflooded	269.5	0.001
Pre-stage flooded vs. post1-stage	285.4	0.001
Pre-stage flooded vs. Post2-stage	309.4	0.001
Pre-stage unflooded vs. post1-stage	151.8	0.011
Pre-stage unflooded vs. post2-stage	170.2	0.003
Post1-stage vs. post2-stage	175.9	0.002
**Overall effect—Ocurrence**	**Wald**	**P**
All categories	19.14	0.001
**Post hoc pairwise comparisons**	**Sum-of-LR statistic**	**P**
Pre-stage flooded vs. pre- stage unflooded	297.1	0.001
Pre- stage flooded vs. post1-stage	297.2	0.001
Pre- stage flooded vs. post2-stage	337.8	0.001
Pre- stage unflooded vs. post1-stage	141.8	0.013
Pre-stage unflooded vs. post2-stage	178.5	0.004
Post1-stage vs. post2-stage	166.0	0.004

Pre-stage flooded = plots that were sampled pre-filling that were flooded; pre-stage unflooded = plots that were sampled pre-filling that were not flooded; post1-stage = plots sampled 1 year after dam filling; post2-stage = plots sampled 4 years after dam filling. Results show deviance table and frequentist probabilities (p) based on 999 bootstrap iterations with PIT-trap resampling. LR means log-likelihood-ratio statistic.

The temporal change in assemblage structure in unflooded plots was mainly caused by gain in species per plot. About 82% of the plots had increases in the number of species from the pre-stage unflooded to post1-stage (p < 0.001; C-B = +) and 65% of the plots had increases in the number of species from the pre-stage unflooded to post2-stage (p = 0.005; C-B = +). Plots had similar abundance per species in pre-stage unflooded and the post1-stage (p = 0.906; C-B = 0) (S6 Table).

The multivariate GLM analyses indicated that there was a temporal change in the frog assemblages of the unflooded plots within 2 km from the flooding area in all comparisons. However, this change was not detected in the assemblages in plots further than 2 km from the flooded area, which showed no statistically significant differences among stages (S7 Table) (S3 Fig).

We used the first two axes of a PCoA analysis to summarize the temporal trajectories of frog assemblages in unflooded plots over the three flooding stages (S4 Fig). Intra-annual changes were more evident along axis 1 of the PCoA, probably resulting from seasonal or weather-induced variation. Displacement along the second axis recorded changes among years, and the general trend for change in that direction was already evident in the year before flooding, indicating that the subsequent changes might not have been due to dam construction.

## Discussion

Overall, our results indicate that the construction of the Santo Antônio hydroelectric had little effect on the number or identity of species in the region. Few species recorded in the pre-stage flooded plots were not found in the pre-stage unflooded plots or in stages after dam filling, and most of these absences were probably due to the vagaries of sampling. This was unexpected as many of the flooded plots were covered in várzea forest, a vegetation association often found to contain unique complements of species of other taxa [17,59]. Nevertheless, the assemblages in flooded areas based on relative abundances were distinct from upland unflooded plots and similar assemblages have not been reconstituted over time in plots near the new banks of the reservoir.

Santo Antônio and Jirau hydroelectric dams flooded 118 km^2^ of várzea forest in the area upstream of the Santo Antônio dam (including the Jirau dam and Bolivian section of the Madeira River). In the Amazon, 83 dams are expected to be built with the potential to affect the floodplains of the Amazon River basin, with the Madeira River sub-basin considered the most threatened in the Amazon [2]. Run-of-the-river dams permanently impact várzea and riparian forests, and less than 1% of Amazonian floodplains in Brazil are in strictly-protected conservation areas, even though the mandatory protected area of 25% gives the impression of extensive floodplain conservation management [60].

Permanent inundation of the floodplains of the major Amazonian rivers could lead to irreparable losses of unique habitats [10,17,59,61]. Assemblages of frogs in várzea and riparian forest have been shown to be distinct from unflooded terra-firme forest [13,62,63]. Várzea forests are periodically inundated by nutrient-rich waters [17] and tend to have more species and higher abundances of frogs than unflooded terra-firme forest. This pattern is also found in other groups of animals and plants [8–13]. However, our analyses did not reveal loss of species due to flooding of most of the várzea forest and this may be because most species can live in riparian areas away from the main river. However, the relative abundances of species were different in the areas that were inundated by the dam, indicating unique ecological processes maintaining assemblages. Similar assemblages may eventually be reconstituted around the edges of the reservoir, but the data up to four years after dam filling does not indicate that this is happening.

Generally, natural or anthropogenic disturbances promote changes in the composition of species [64,65]. In unflooded areas around the Santo Antônio dam, frog assemblages showed constant temporal changes in species relative abundances and the number of species per plot, especially for plots within two kilometers of the new reservoir bank, and these changes had not stopped four years after dam filling. The temporal change in species composition was accompanied by a reduction in the relative abundance of frogs in most plots, but the number of species increased. The margins of a newly formed reservoir generally do not replicate the same alluvial habitats that previously existed [66,67]. However, there is some evidence that the frog assemblages in the unflooded area were changing even before dam construction, so we cannot be sure whether these changes were caused by the dam filling. Extrapolation in the rarefaction analyses indicates that the total number of species (gamma diversity) might not differ among flooding stages, even though there are differences in alpha and beta diversity.

Contrary to our expectations based on data from mega dams that alter extensive areas of native vegetation [28,68], the number of species increased in 65% of the plots four years after dam filling, and the changes were greatest within two kilometers of the new bank of the reservoir. The increase in number of species in areas adjacent to those flooded by the dam may be an example of the dam’s extended effect [66,69]. After reservoir filling, the animals displaced by the flooding move to the nearest remaining areas, which may have increased the number of species recorded per plot in the non-flooded areas. In the period of community restructuration after a disturbance, some populations may decrease while others can occupy the newly formed environments [70,71]. The effects of environmental impacts is almost immediate in some cases, but often it takes a considerable amount of time for declining populations to disappear following environmental perturbations [64].

Despite the changes in relative abundances of species in assemblages, dam filling did not extinguish many species, as is expected for large hydroelectric projects ([72]). Most of the species recorded in the area to be inundated before flooding were also recorded post flooding, and 40% of species were found in all flooding stages. A few species of frogs (N = 7) that were recorded in the pre-stage flooded plots may have disappeared from the area around the Santo Antônio hydroelectric dam. However, six of the seven species not recorded after dam filling occurred in only one plot and may have been absent from the post-filling surveys simply because of the vagaries of sampling. The only species found in more than one plot that was not captured subsequently, *Adenomera* gr. *marmoratus* sp1, occurred in the Morrinhos sampling module that was completely inundated. Surveys specifically for species that occurred in the Morrinhos module revealed this species to still be present on the new banks of the reservoir. This indicates that hydroelectric plants with run-of-the-river turbines may not eliminate species locally if areas with native vegetation are conserved around the new banks of the river.

The limited data collected before dam filling showed the same temporal trajectory in assemblage composition in the unflooded areas as the stages after construction of the dam. So, it is possible that the changes are related to some long-term phenomenon unrelated to the dam, and it is not possible to discount this possibility with the small number of sample dates before the dam was closed.

The faunal surveys associated with the environmental-impact evaluations for the Santo Antônio hydroelectric dam were among the most intensive, well planned and long-term ever carried out in Brazil and studies of other faunal groups have already been published, including papers on bats, fish and trees [18,21,72,73]. However, understanding long-term effects on biodiversity requires long-term studies and, in the case of large-scale infrastructure projects, future research should start well before dam construction so that natural fluctuations in species densities can be documented. The lack of understanding by the controlling authorities of the long-term effects of landscape changes, such as water-table rises, means that they also do not require studies covering appropriate periods post construction. There is evidence of long-term changes in the structure of the frog assemblages around the Santo Antônio dam, with no indication of return to the initial compositions. Long-term monitoring after project installation with less frequent sampling would allow studies over the longer periods that are often associated with relaxation of biotic communities [67].

In summary, few, if any, species were lost from the area as a result of the Santo Antonio dam. Some assemblages with unique combinations which occurred in the areas that were flooded were not recomposed on the new banks of the reservoir during the study period. There was evidence that the dam affected the compositions of frog assemblages in unflooded areas, especially those closest to the reservoir. The mean number of species increased over time in unflooded plots, without substitution of the original species complements. However, limited evidence indicated that those changes may have been happening in unflooded areas before dam closure. Stronger conclusions about these changes in species composition would be possible using a BACI design [74]. We recommend that environmental authorities require long-term monitoring and BACI designs in future environmental-impact studies.

## Supporting information

S1 Fig Sample-based rarefaction and extrapolation for combinations of flooding and time since filling of the Santo Antônio dam on the Madeira River, southwestern Brazilian Amazonia. with 95% unconditional confidence intervals (shaded area, bootstrap with 1,000 replications). Each of the curves is extrapolated up to the maximum sample size of 60 sample units.
(TIF)

S2 Fig Plots of relative abundance (log [x + 1] transformed relative abundance values to better visualization) of frogs in relation to flooding and time since filling of the Santo Antônio dam on the Madeira River, southwestern Brazilian Amazonia. green = plots that were sampled pre-filling that were flooded; blue = plots that were sampled pre-filling that were not flooded; black = plots sampled 1 year after dam filling; red = plots sampled 4 years after dam filling.
(TIF)

S3 Fig First two axes of a principal coordinates analysis (PCoA) based on relative abundance in both near (≤ 2 km) (A) and distant (> 2 km) (B) plots showing the 95% confidence ellipses for the centroids of samples in relation to flooding and time since filling of the Santo Antônio dam on the Madeira River, southwestern Brazilian Amazonia. blue = plots that were sampled pre-filling that were not flooded; black = plots sampled 1 year after dam filling; red = plots sampled 4 years after dam filling.
(TIF)

S4 Fig First two axes of a principal coordinates ordination (PCoA) of frog species composition showing the 95% confidence ellipses for the centroids of samples in plots in relation to the flooding and time since filling of the Santo Antônio dam on the Madeira River, southwestern Brazilian Amazonia. blue = plots that were sample pre-filling that were not flooded, black = plots sampled 2 years after dam filling, red = plots sampled 4 years after dam filling. Numbers indicate temporal trajectories. Dotted lines indicate change of flooding period. All data were grouped in each survey period resulting in nine points based on the same 39 plots sampled in each period.
(TIF)

S5 Fig Distribution of plots in the multivariate space defined by the best-fit latent variables based on relative abundance (A) with a negative-binomial distribution and occurrence (B) with a binomial distribution of frog species showing the 95% confidence ellipses for the centroids of samples in plots in relation to the flooding and time since filling of the Santo Antônio dam on the Madeira River, southwestern Brazilian Amazonia. green = plots that were sampled pre-filling that were flooded; blue = plots that were sampled pre-filling that were not flooded; black = plots sampled 1 year after dam filling; red = plots sampled 4 years after dam filling.
(TIF)

S6 Fig Distribution of plots in the multivariate space defined by the best-fit latent variables based on relative abundance with a negative-binomial distribution in both near (≤ 2 km) (A) and distant (> 2 km) (B) plots showing the 95% confidence ellipses for the centroids of samples in relation to the flooding and time since filling of the Santo Antônio dam on the Madeira River, southwestern Brazilian Amazonia. blue = plots that were sampled pre-filling that were not flooded; black = plots sampled 1 year after dam filling; red = plots sampled 4 years after dam filling.
(TIF)

S1 Table Summary of flooding, time since dam filling, distance from river and coordinates of the 66 plots sampled around the Santo Antônio hydroelectric dam, Western Amazonia, Brazil. Pre-stage flooded = plots that were sampled pre-filling that were flooded; pre-stage unflooded = plots that were sample pre-filling that were not flooded; post1-stage = plots sampled 1 year after reservoir filling; post2-stage = plots sampled 4 years after reservoir filling.
(XLSX)

S2 Table Number of species recorded, median of abundance and number of plots with records of anuran species sampled in relation to flooding and time since filling of the Santo Antônio hydroelectric dam on the Madeira River, Western Amazonia, Brazil. n plots = number of plots with species records; total plots = total number of plots surveyed; pre-stage flooded = Plots that were sampled pre-filling that were flooded; pre-stage unflooded = plots that were sample pre-filling that were not flooded; post1 stage = plots sampled 1 year after reservoir filling; post2-stage = plots sampled 4 years after reservoir filling.
(XLSX)

S3 Table Manyglm analysis examining the association between the structure of frog assemblages with only abundant species (at least 5% abundance and 4% of plots in our sample) recorded in flooded and unflooded plots around the Madeira River, southwestern Brazilian Amazonia. Pre-stage flooded = plots that were sampled pre-filling that were flooded; pre-stage unflooded = plots that were sampled pre-filling that were not flooded; post1-stage = plots sampled 1 year after dam filling; post2-stage = plots sampled 4 years after dam filling. Results show deviance table and frequentist probabilities (p) based on 999 bootstrap iterations with PIT-trap resampling. LR means log-likelihood-ratio statistic.
(DOCX)

S4 Table Manyglm analysis examining the association between the structure of assemblages with only abundant species (at least 8% abundance and 5% of plots in our sample) recorded in flooded and unflooded plots around the Madeira River, southwestern Brazilian Amazonia. Pre-stage flooded = plots that were sampled pre-filling that were flooded; pre-stage unflooded = plots that were sampled pre-filling that were not flooded; post1-stage = plots sampled 1 year after dam filling; post2-stage = plots sampled 4 years after dam filling. Results show deviance table and frequentist probabilities (p) based on 999 bootstrap iterations with PIT-trap resampling.
(DOCX)

S5 Table Manyglm analysis examining the association between the structure of assemblages of species considered rare in the sampling (only records with up to 5% abundance and 4% of plots in our sample) in flooded and unflooded plots around the Madeira River, southwestern Brazilian Amazonia. Pre-stage flooded = plots that were sampled pre-filling that were flooded; pre-stage unflooded = plots that were sampled pre-filling that were not flooded; post1-stage = plots sampled 1 year after dam filling; post2-stage = plots sampled 4 years after dam filling. Results show deviance table and frequentist probabilities (p) based on 999 bootstrap iterations with PIT-trap resampling.
(DOCX)

S6 Table Tests for differences in temporal beta-diversity indices (TBI) for the structure of frog assemblages between the flooding stages in unflooded plots around the Santo Antônio reservoir on the Madeira River, southwestern Brazilian Amazonia, Brazil. These analyses include only plots surveyed during all flooding stages. N_plots+ is the number of plots with gains in number of species or abundance of species; N_plots- is the number of plots with losses in number of species or abundance of species and N_plots0 is the number of plots without changes in number of species or abundance of species. Pre-stage unflooded = plots that were sample pre-filling that were not flooded; post1-stage = plots sampled 1 year after reservoir filling; post2-stage = plots sampled 4 years after reservoir filling. *p* represents the frequentist probability of a difference between the *B* and *C* statistics.
(DOCX)

S7 Table Manyglm analysis for pairwise tests of differences in the structure of frog assemblages less than 2 km and between 2 km and 5 km distant from the flood margin between the flooding stages in unflooded plots of the Santo Antônio reservoir in the Madeira River, southwestern Brazilian Amazonia, Brazil. Pre-stage unflooded = plots that were sample pre-filling that were not flooded; post1-stage = plots sampled 1 year after reservoir filling; post2-stage = plots sampled 4 years after reservoir filling. Results show deviance table and frequentist probabilities (p) values based on 999 bootstrap iterations with PIT-trap resampling. LR means log-likelihood-ratio statistic.
(DOCX)
