# Supplementary figures and images for: Impacts of an Amazonian hydroelectric dam on frog assemblages
Source: PLoS One. 2021 Jun 17;16(6):e0244580. doi: 10.1371/journal.pone.0244580 (PMC8211156; doi:10.1371/journal.pone.0244580)

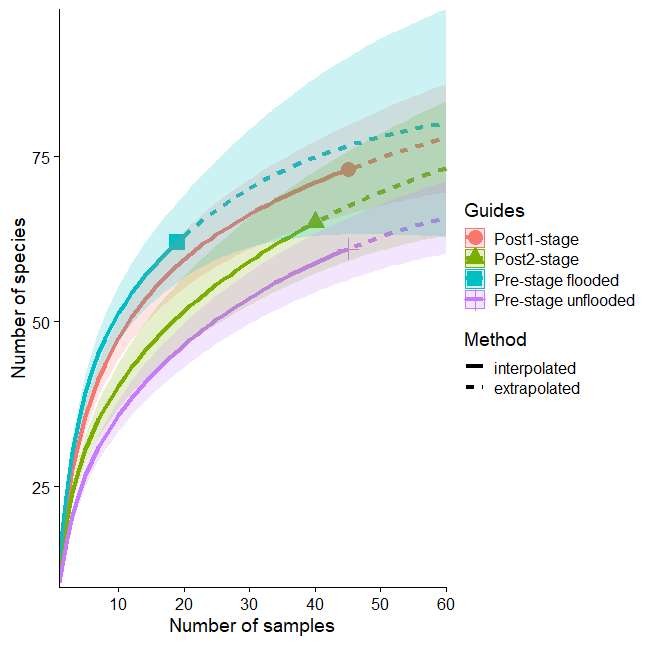

Supplement: S1 Fig — with 95% unconditional confidence intervals (shaded area, bootstrap with 1,000 replications). Each of the curves is extrapolated up to the maximum sample size of 60 sample units. (TIF) [file pone.0244580.s001.tif]

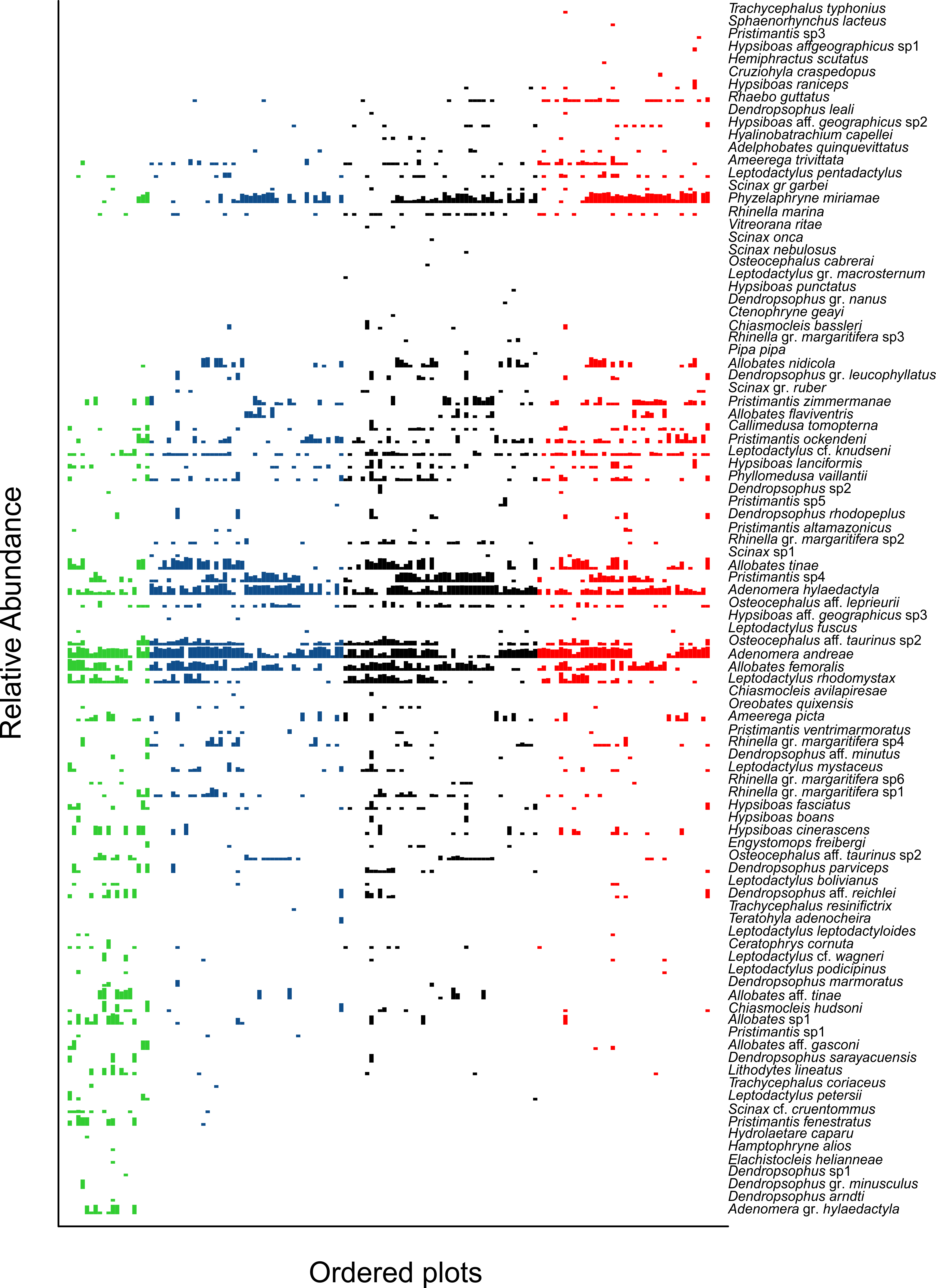

Supplement: S2 Fig — green = plots that were sampled pre-filling that were flooded; blue = plots that were sampled pre-filling that were not flooded; black = plots sampled 1 year after dam filling; red = plots sampled 4 years after dam filling. (TIF) [file pone.0244580.s002.tif]

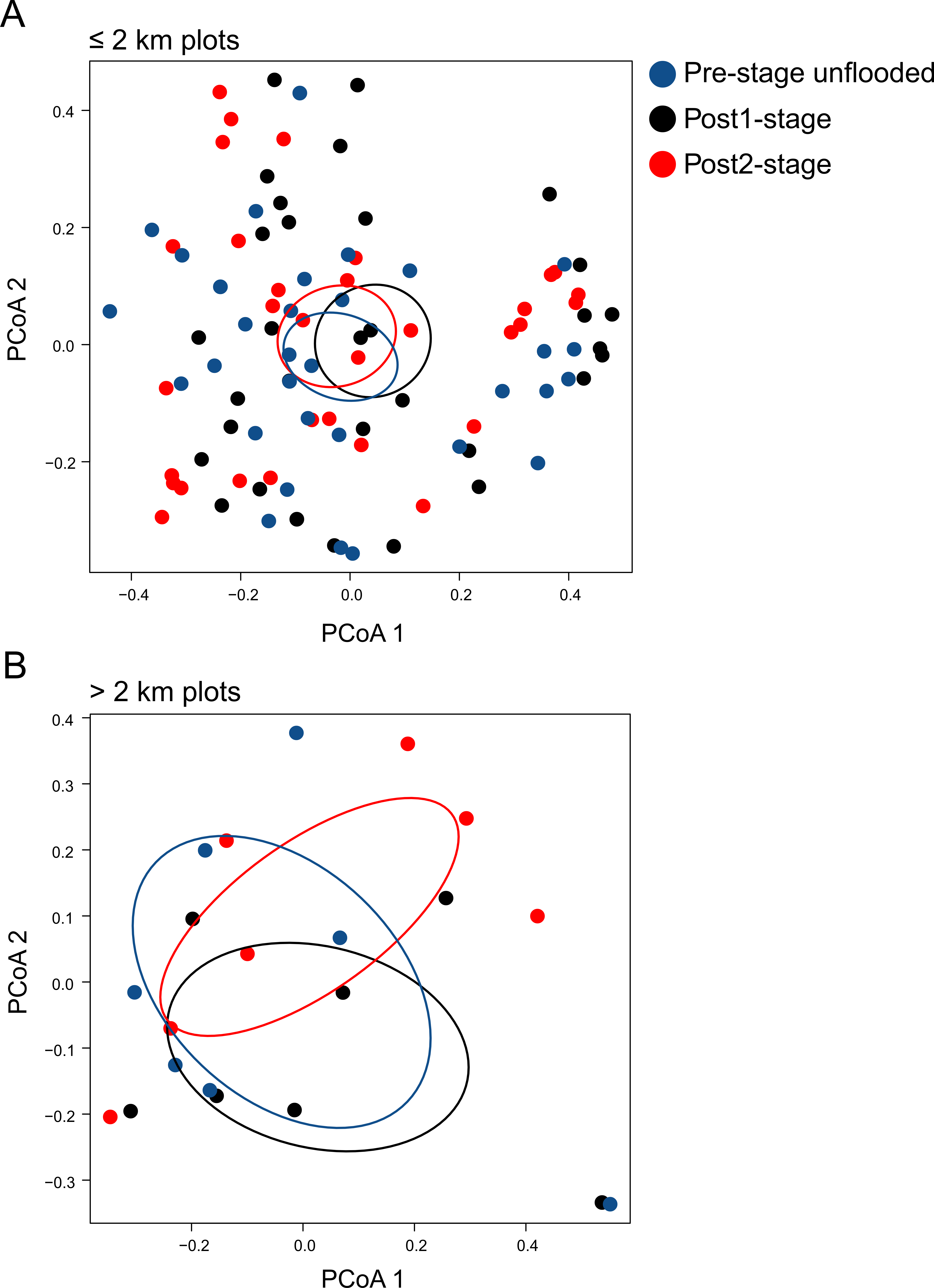

Supplement: S3 Fig — blue = plots that were sampled pre-filling that were not flooded; black = plots sampled 1 year after dam filling; red = plots sampled 4 years after dam filling. (TIF) [file pone.0244580.s003.tif]

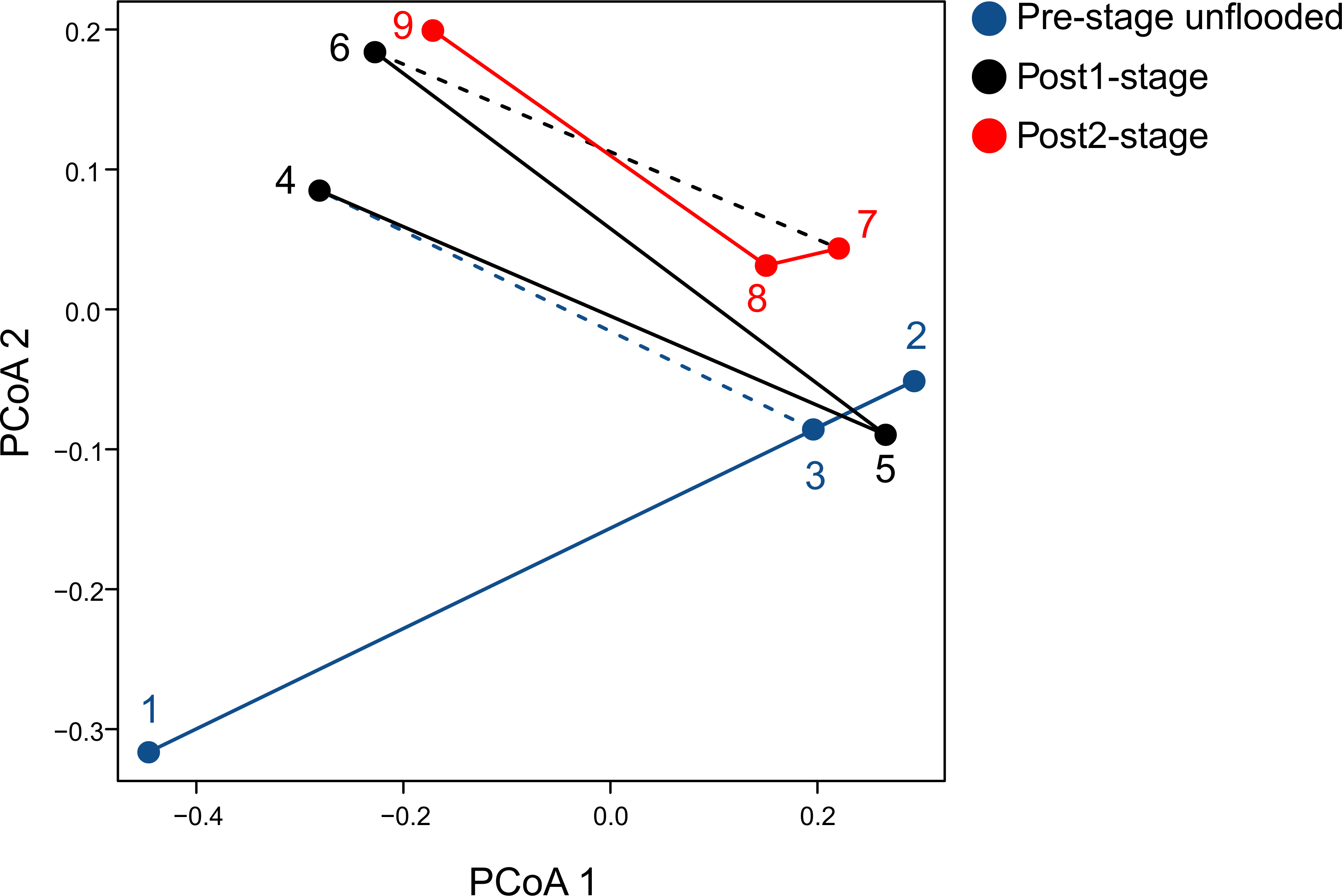

Supplement: S4 Fig — blue = plots that were sample pre-filling that were not flooded, black = plots sampled 2 years after dam filling, red = plots sampled 4 years after dam filling. Numbers indicate temporal trajectories. Dotted lines indicate change of flooding period. All data were grouped in each survey period resulting in nine points based on the same 39 plots sampled in each period. (TIF) [file pone.0244580.s004.tif]

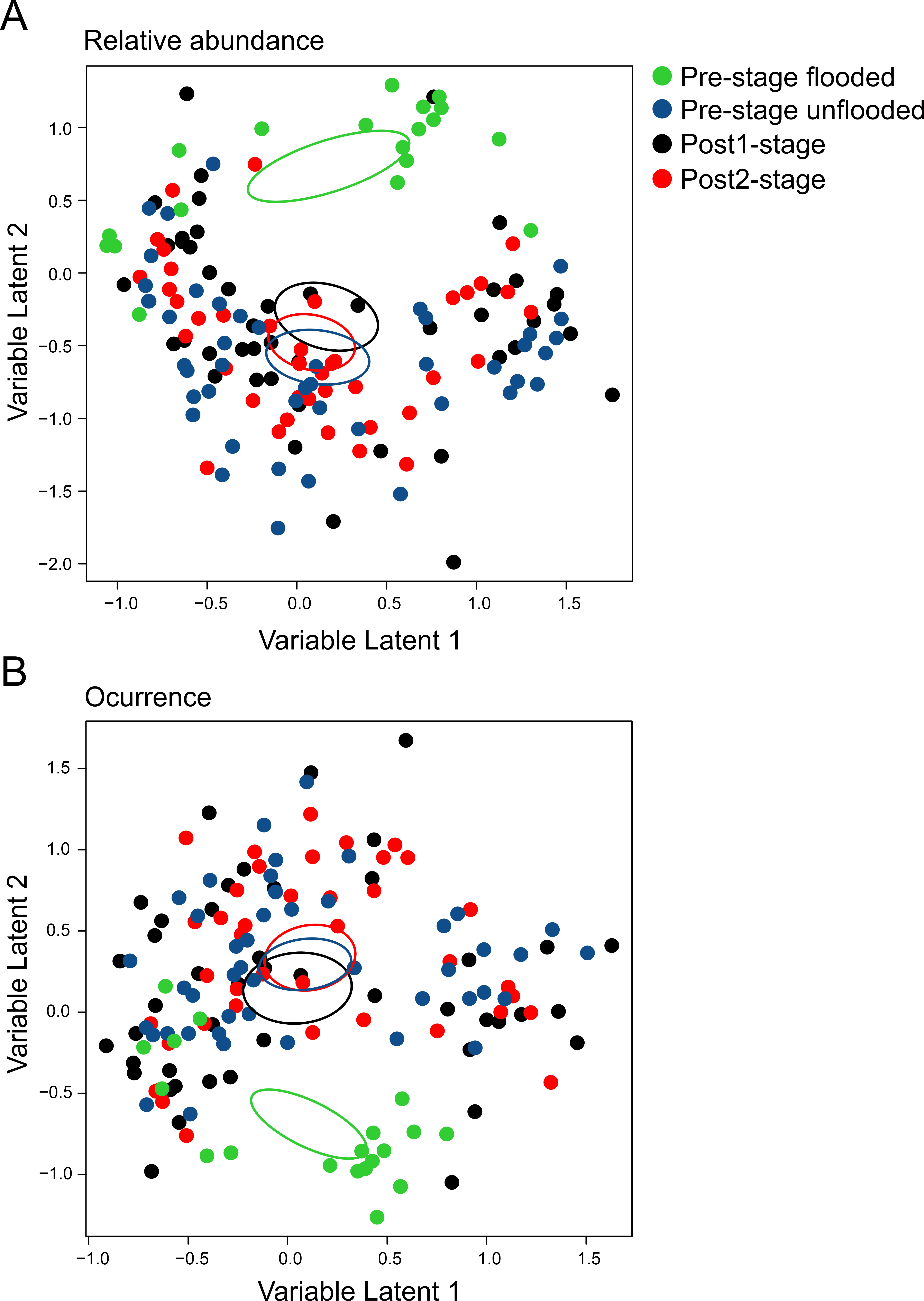

Supplement: S5 Fig — green = plots that were sampled pre-filling that were flooded; blue = plots that were sampled pre-filling that were not flooded; black = plots sampled 1 year after dam filling; red = plots sampled 4 years after dam filling. (TIF) [file pone.0244580.s005.tif]

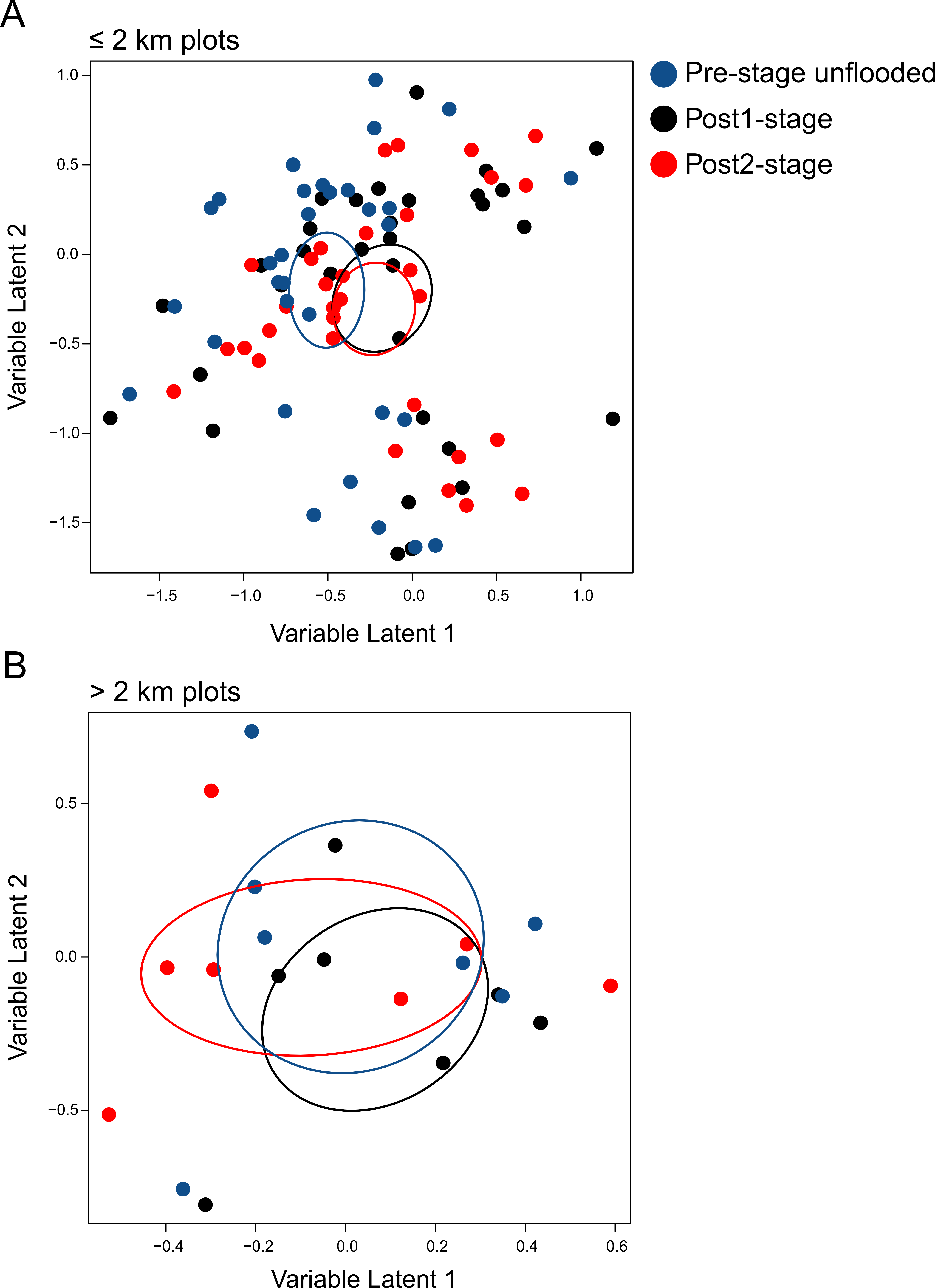

Supplement: S6 Fig — blue = plots that were sampled pre-filling that were not flooded; black = plots sampled 1 year after dam filling; red = plots sampled 4 years after dam filling. (TIF) [file pone.0244580.s006.tif]
