# Supplementary material for: Impacts of an Amazonian hydroelectric dam on frog assemblages
Source: PLoS One. 2021 Jun 17;16(6):e0244580. doi: 10.1371/journal.pone.0244580 (PMC8211156; doi:10.1371/journal.pone.0244580)
Supplement: S3 Table — Pre-stage flooded = plots that were sampled pre-filling that were flooded; pre-stage unflooded = plots that were sampled pre-filling that were not flooded; post1-stage = plots sampled 1 year after dam filling; post2-stage = plots sampled 4 years after dam filling. Results show deviance table and frequentist probabilities (p) based on 999 bootstrap iterations with PIT-trap resampling. LR means log-likelihood-ratio statistic. (DOCX) [file pone.0244580.s009.docx]

**S3 Table. Manyglm analysis examining the association between the structure of frog assemblages with only abundant species (at least 5% abundance and 4% of plots in our sample) recorded in flooded and unflooded plots around the Madeira River, southwestern Brazilian Amazonia.** Pre-stage flooded = plots that were sampled pre-filling that were flooded; pre-stage unflooded = plots that were sampled pre-filling that were not flooded; post1-stage = plots sampled 1 year after dam filling; post2-stage = plots sampled 4 years after dam filling. Results show deviance table and frequentist probabilities (p) based on 999 bootstrap iterations with PIT-trap resampling. LR means log-likelihood-ratio statistic.

| **Overall effect – Abundance** | **Wald** | **p** |
| --- | --- | --- |
| All treatments | 20.22 | 0.001 |
| **Post hoc pairwise comparisons** | **Sum-of-LR statistic** | **p** |
| Pre-stage flooded vs. pre-stage unflooded | 247.2 | 0.001 |
| Pre-stage flooded vs. post1-stage | 253.9 | 0.001 |
| Pre-stage flooded vs. post2-stage | 284.2 | 0.001 |
| Pre-stage unflooded vs. post1-stage | 126.0 | 0.003 |
| Pre-stage unflooded vs. post2-stage | 152.2 | 0.010 |
| Post1-stage vs. post2-stage | 149.6 | 0.004 |
|  |  |  |
| **Overall effect – Ocurrence** | **Wald** | **p** |
| All treatments | 19.45 | 0.001 |
| **Post hoc pairwise comparisons** | **Sum-of-LR statistic** | **p** |
| Pre-stage flooded vs. pre-stage unflooded | 280.9 | 0.001 |
| Pre-stage flooded vs. post1-stage | 277.4 | 0.001 |
| Pre-stage flooded vs. post2-stage | 319.3 | 0.001 |
| Pre-stage unflooded vs. post1-stage | 129.2 | 0.013 |
| Pre-stage unflooded vs. post2-stage | 166.8 | 0.005 |
| Post1-stage vs. post2-stage | 147.9 | 0.011 |
