# Supplementary material for: Impacts of an Amazonian hydroelectric dam on frog assemblages
Source: PLoS One. 2021 Jun 17;16(6):e0244580. doi: 10.1371/journal.pone.0244580 (PMC8211156; doi:10.1371/journal.pone.0244580)
Supplement: S4 Table — Pre-stage flooded = plots that were sampled pre-filling that were flooded; pre-stage unflooded = plots that were sampled pre-filling that were not flooded; post1-stage = plots sampled 1 year after dam filling; post2-stage = plots sampled 4 years after dam filling. Results show deviance table and frequentist probabilities (p) based on 999 bootstrap iterations with PIT-trap resampling. (DOCX) [file pone.0244580.s010.docx]

**S4 Table: Manyglm analysis examining the association between the structure of assemblages with only abundant species (at least 8% abundance and 5% of plots in our sample) recorded in flooded and unflooded plots around the Madeira River, southwestern Brazilian Amazonia.** Pre-stage flooded = plots that were sampled pre-filling that were flooded; pre-stage unflooded = plots that were sampled pre-filling that were not flooded; post1-stage = plots sampled 1 year after dam filling; post2-stage = plots sampled 4 years after dam filling. Results show deviance table and frequentist probabilities (p) based on 999 bootstrap iterations with PIT-trap resampling.

| **Overall effect – Abundance** | **Wald** | **p** |
| --- | --- | --- |
| All treatments | 19.38 | 0.001 |
| **Post hoc pairwise comparisons** | **Sum-of-LR statistic** | **p** |
| Pre-stage flooded vs. pre-stage unflooded | 203.3 | 0.001 |
| Pre-stage flooded vs. post1-stage | 217.4 | 0.001 |
| Pre-stage flooded vs. post2-stage | 250.6 | 0.001 |
| Pre-stage unflooded vs. post1-stage | 110.9 | 0.001 |
| Pre-stage unflooded vs. post2-stage | 138.8 | 0.011 |
| Post1-stage vs. post2-stage | 128.2 | 0.002 |
|  |  |  |
| **Overall effect – Ocurrence** | **Wald** | **p** |
| All treatments | 18.42 | 0.001 |
| **Post hoc pairwise comparisons** | **Sum-of-LR statistic** | **p** |
| Pre-stage flooded vs. pre-stage unflooded | 232.5 | 0.001 |
| Pre-stage flooded vs. post1-stage | 227.1 | 0.001 |
| Pre-stage flooded vs. post2-stage | 286.4 | 0.001 |
| Pre-stage unflooded vs. post1-stage | 102.8 | 0.001 |
| Pre-stage unflooded vs. post2-stage | 148.5 | 0.011 |
| Post1-stage vs. post2-stage | 119.6 | 0.018 |
