# Supplementary material for: Impacts of an Amazonian hydroelectric dam on frog assemblages
Source: PLoS One. 2021 Jun 17;16(6):e0244580. doi: 10.1371/journal.pone.0244580 (PMC8211156; doi:10.1371/journal.pone.0244580)
Supplement: S5 Table — Pre-stage flooded = plots that were sampled pre-filling that were flooded; pre-stage unflooded = plots that were sampled pre-filling that were not flooded; post1-stage = plots sampled 1 year after dam filling; post2-stage = plots sampled 4 years after dam filling. Results show deviance table and frequentist probabilities (p) based on 999 bootstrap iterations with PIT-trap resampling. (DOCX) [file pone.0244580.s011.docx]

**S5 Table: Manyglm analysis examining the association between the structure of assemblages of species considered rare in the sampling (only records with up to 5% abundance and 4% of plots in our sample) in flooded and unflooded plots around the Madeira River, southwestern Brazilian Amazonia.** Pre-stage flooded = plots that were sampled pre-filling that were flooded; pre-stage unflooded = plots that were sampled pre-filling that were not flooded; post1-stage = plots sampled 1 year after dam filling; post2-stage = plots sampled 4 years after dam filling. Results show deviance table and frequentist probabilities (p) based on 999 bootstrap iterations with PIT-trap resampling.

| **Overall effect – Abundance** | **Wald** | **p** |
| --- | --- | --- |
| All treatments | 2.264 | 0.185 |
| **Post hoc pairwise comparisons** | **Sum-of-LR statistic** | **p** |
| Pre-stage flooded vs. pre-stage unflooded | 22.29 | 0.098 |
| Pre-stage flooded vs. post1-stage | 31.49 | 0.022 |
| Pre-stage flooded vs. post2-stage | 25.14 | 0.098 |
| Pre-stage unflooded vs. post1-stage | 25.76 | 0.098 |
| Pre-stage unflooded vs. post2-stage | 17.95 | 0.098 |
| Post1-stage vs. post2-stage | 26.33 | 0.098 |
|  |  |  |
| **Overall effect – Ocurrence** | **Wald** | **p** |
| All treatments | 0.552 | 0.871 |
| **Post hoc pairwise comparisons** | **Sum-of-LR statistic** | **p** |
| Pre-stage flooded vs. pre-stage unflooded | 16.22 | 0.076 |
| Pre-stage flooded vs. post1-stage | 19.78 | 0.076 |
| Pre-stage flooded vs. post2-stage | 18.53 | 0.076 |
| Pre-stage unflooded vs. post1-stage | 12.58 | 0.122 |
| Pre-stage unflooded vs. post2-stage | 11.69 | 0.122 |
| Post1-stage vs. post2-stage | 18.10 | 0.076 |
