# Supplementary material for: Impacts of an Amazonian hydroelectric dam on frog assemblages
Source: PLoS One. 2021 Jun 17;16(6):e0244580. doi: 10.1371/journal.pone.0244580 (PMC8211156; doi:10.1371/journal.pone.0244580)
Supplement: S6 Table — These analyses include only plots surveyed during all flooding stages. N_plots+ is the number of plots with gains in number of species or abundance of species; N_plots- is the number of plots with losses in number of species or abundance of species and N_plots0 is the number of plots without changes in number of species or abundance of species. Pre-stage unflooded = plots that were sample pre-filling that were not flooded; post1-stage = plots sampled 1 year after reservoir filling; post2-stage = plots sampled 4 years after reservoir filling. p represents the frequentist probability of a difference between the B and C statistics. (DOCX) [file pone.0244580.s012.docx]

| **S6 Table. Tests for differences in temporal beta-diversity indices (TBI) for the structure of frog assemblages between the flooding stages in unflooded plots around the Santo Antônio reservoir on the Madeira River, southwestern Brazilian Amazonia, Brazil.** These analyses include only plots surveyed during all flooding stages. N_plots+ is the number of plots with gains in number of species or abundance of species; N_plots- is the number of plots with losses in number of species or abundance of species and N_plots0 is the number of plots without changes in number of species or abundance of species. Pre-stage unflooded = plots that were sample pre-filling that were not flooded; post1-stage = plots sampled 1 year after reservoir filling; post2-stage = plots sampled 4 years after reservoir filling. *p* represents the frequentist probability of a difference between the *B* and *C* statistics.   \| **Post hoc pairwise comparisons** \| **B (losses)** \| **C (gains)** \| **C-B (change)** \| **N_plots-** \| **N_plots+** \| **n_plots0** \| **p** \| \| --- \| --- \| --- \| --- \| --- \| --- \| --- \| --- \| \| Pre-stage unflooded vs. post1-stage – Abundance \| 0.22 \| 0.22 \| 0 \| 23 \| 19 \| 3 \| 0.906 \| \| Pre-stage unflooded vs. post2-stage - Abundance \| 0.27 \| 0.23 \| - \| 25 \| 15 \| 0 \| 0.198 \| \| Pre-stage unflooded vs. post1-stage – Ocurrence \| 0.12 \| 0.27 \| + \| 3 \| 37 \| 5 \| < 0.001 \| \| Pre-stage unflooded vs. post2-stage - Ocurrence \| 0.17 \| 0.26 \| + \| 10 \| 26 \| 4 \| 0.005 \| |
| --- | --- | --- | --- | --- | --- | --- | --- | --- | --- | --- | --- | --- | --- | --- | --- | --- | --- | --- | --- | --- | --- | --- | --- | --- | --- | --- | --- | --- | --- | --- | --- | --- | --- | --- | --- | --- | --- | --- | --- | --- |
