# Supplementary material for: Impacts of an Amazonian hydroelectric dam on frog assemblages
Source: PLoS One. 2021 Jun 17;16(6):e0244580. doi: 10.1371/journal.pone.0244580 (PMC8211156; doi:10.1371/journal.pone.0244580)
Supplement: S7 Table — Pre-stage unflooded = plots that were sample pre-filling that were not flooded; post1-stage = plots sampled 1 year after reservoir filling; post2-stage = plots sampled 4 years after reservoir filling. Results show deviance table and frequentist probabilities (p) values based on 999 bootstrap iterations with PIT-trap resampling. LR means log-likelihood-ratio statistic. (DOCX) [file pone.0244580.s013.docx]

**S7 Table. Manyglm analysis for pairwise tests of differences in the structure of frog assemblages less than 2 km and between 2 km and 5 km distant from the flood margin between the flooding stages in unflooded plots of the Santo Antônio reservoir in the Madeira River, southwestern Brazilian Amazonia, Brazil.** Pre-stage unflooded = plots that were sample pre-filling that were not flooded; post1-stage = plots sampled 1 year after reservoir filling; post2-stage = plots sampled 4 years after reservoir filling. Results show deviance table and frequentist probabilities (p) values based on 999 bootstrap iterations with PIT-trap resampling. LR means log-likelihood-ratio statistic.

| **Pairwise comparisons** | **Sum-of-LR statistic** | **p** |
| --- | --- | --- |
| **until 2 km** |  |  |
| Pre-stage unflooded vs. post1-stage | 133.3 | 0.036 |
| Pre-stage unflooded vs. post2-stage | 135.1 | 0.034 |
| Post1-stage vs. post2-stage | 132.5 | 0.036 |
| **Between 2 km and 5 km** | **Sum-of-LR statistic** | **p** |
| Pre-stage unflooded vs. post1-stage | 63.30 | 0.239 |
| Pre-stage unflooded vs. post2-stage | 82.51 | 0.209 |
| Post1-stage vs. post2-stage | 78.56 | 0.209 |
